# Non-linear mapping for exploratory data analysis in functional genomics

**DOI:** 10.1186/1471-2105-6-13

**Published:** 2005-01-20

**Authors:** Francisco Azuaje, Haiying Wang, Alban Chesneau

**Affiliations:** 1 School of Computing and Mathematics, University of Ulster, BT37 0QB, UK; 2 Molecular Genetics Institute, CNRS UMR5535, 1919, route de Mende, 34293 Montpellier, France

## Abstract

**Background:**

Several supervised and unsupervised learning tools are available to classify functional genomics data. However, relatively less attention has been given to exploratory, visualisation-driven approaches. Such approaches should satisfy the following factors: Support for intuitive cluster visualisation, user-friendly and robust application, computational efficiency and generation of biologically meaningful outcomes. This research assesses a relaxation method for non-linear mapping that addresses these concerns. Its applications to gene expression and protein-protein interaction data analyses are investigated

**Results:**

Publicly available expression data originating from leukaemia, round blue-cell tumours and Parkinson disease studies were analysed. The method distinguished relevant clusters and critical analysis areas. The system does not require assumptions about the inherent class structure of the data, its mapping process is controlled by only one parameter and the resulting transformations offer intuitive, meaningful visual displays. Comparisons with traditional mapping models are presented. As a way of promoting potential, alternative applications of the methodology presented, an example of exploratory data analysis of interactome networks is illustrated. Data from the *C. elegans *interactome were analysed. Results suggest that this method might represent an effective solution for detecting key network hubs and for clustering biologically meaningful groups of proteins.

**Conclusion:**

A relaxation method for non-linear mapping provided the basis for visualisation-driven analyses using different types of data. This study indicates that such a system may represent a user-friendly and robust approach to exploratory data analysis. It may allow users to gain better insights into the underlying data structure, detect potential outliers and assess assumptions about the cluster composition of the data.

## Background

Systems biology is a data- and knowledge-driven discipline, which heavily relies on automated tools to support the generation and validation of hypotheses. Such tasks aim to provide novel and meaningful views of the functional relationships between biological components at different complexity levels. Over the past seven years hundreds of methods have been reported to analyse these data, with an emphasis on gene expression data classification [1,2]. More recently, the analysis of gene regulatory and protein-protein networks has started to attract contributions from computer and physical sciences [3-5]. All of these tasks are linked by a need for comparing, classifying and visualising information.

The ever-increasing number and sophistication of techniques may represent an obstacle to achieve more meaningful and rigorous data analysis and discovery tasks. One important problem is that users may not have the time and knowledge required to adequately understand the dynamics and operation of several tools. These deficiencies have been reflected, for example, in a lack of sound practices for assessing the statistical significance of results and for selecting the most suitable data sets and classification models [6,7]. On the other hand, the emergence of multiple data sets and prediction models represents an opportunity for developing an integrative data mining paradigm, which is already significantly improving several predictive tasks in systems biology [5,8,9].

The problem domains mentioned above have mainly concentrated on the application of statistical and machine learning models for classification tasks. Emphasis has been placed on the development of supervised and unsupervised classification methods [2,10,11], as well as on the application of statistical tools for assessing the quality of classification results [12,13]. Relatively fewer efforts have been reported on *data visualisation techniques *to support *exploratory analysis*. It has been shown that information visualisation techniques may support predictive data mining applications, including data clustering [14-16]. These tasks should complement each other in order to achieve higher levels of knowledge integration and understanding. Furthermore, visualisation-based exploratory methods may support: a) the identification of key patterns in the data, and b) the selection of the most adequate models for data pre-processing and/or classification. The former task refers to the recognition of key groups of data, outliers and features based on computationally-inexpensive, user-friendly and robust analyses. Its outcomes may offer guidance to conduct the latter task by gaining a better insight into the high-level structure and relationships found in the data. Such an exploratory, visualisation-based approach may generate useful alternative views for supporting a more intelligent and meaningful application of classification models.

One of the traditional approaches to functional genomics information visualisation has been the application of clustering-based visualisation techniques. Such an approach mainly consists of two steps: a) the implementation of a clustering algorithm, and b) the display of the obtained clusters. The resulting clusters may be visualised by generating, for example, dendrograms [16], other hierarchical structures [17] and maps [14,18,19], which highlight or summarise similarity relationships between groups of data. Clustering-based visualisation has become a fundamental tool for analysing gene and protein expression data. Different variations of hierarchical clustering, *Kohonen Self Organising Maps *(SOM) and *Self-Adaptive Neural Networks *(SANN) are relevant examples of techniques belonging to this approach. Their capabilities and applications have been widely reported [2,15,20]. They have been successfully tested on several classification and decision support problems. However, its application to visualisation-driven exploratory analysis is limited by several problems: Many of these techniques are not capable of explicitly and automatically detecting cluster boundaries; some of them are critically sensitive to several learning parameters that need to be selected by the user; some of these solutions were not originally designed to tackle cluster-based visualisation tasks of massive collections of data described by several thousands attributes; and they traditionally require assumptions about the inherent structure of the data, which may not be always possible in exploratory data analyses. Clustering visualisation has also become an important task for the analysis of protein-protein interaction networks. Clustering is a fundamental mathematical property of networks, which allows the identification of key connectivity patterns. Such patterns may be associated with significant functional behaviours and modularity [3]. Moreover, it allows researchers to identify relevant areas for further statistical or experimental analyses. For instance, hierarchical clustering of network nodes (proteins) has been applied to detect functional modules in *S. cerevisiae *[3]. Each node may be represented by a vector of connectivity values that reflects the node's interactions with other network members. Graph theoretic approaches have also been applied to detect significant clusters of interconnected proteins [21].

Another important data visualisation approach, which may be applied to clustering-based analysis, comprises the application of *non-linear mapping techniques*. They are based on the idea of transforming the original, *n*-dimensional input space into a reduced, *m*-dimensional one, where *m*<*n*. These methods are also known as *non-linear projection methods *or *multidimensional scaling *(MDS) methods [22]. They mainly aim to optimize a function, *M*, which reflects key aspects of the distance structure of the original *n*-dimensional space. Thus, these methods aim to preserve such global properties in the transformed, *m*-dimensional space. This principle has been followed by several models including those proposed by Kruskal [23,24] and Sammon [25]. *Principal component analysis *(PCA) [26] is another relevant technique for reducing *n*-dimensional data. In this case the resulting transformation accounts for the greatest variation of the original space, but without preserving the distances observed between the points in the *n*-dimensional space. MDS, including Sammon's mapping, applications to gene expression analysis have been reported, which highlight their advantages for supporting the detection of clusters [27]. PCA may not be directly used to visualise clusters. But it may be applied as a pre-processing procedure, and its resulting components may be used as inputs to clustering and supervised classification models [28].

Although widely investigated techniques, such as SOM and Sammon's mapping, are usually useful to visualise clusters of high-dimensional data, they present several limitations. For example, the SOM may be highly sensitive to its training parameters, which have to be defined by the user. It also requires the user to define map topologies, and it does not provide automated mechanisms for cluster boundary detection. Its limitations for data exploratory analysis, particularly in relation to data topology preservation, have been stressed in [29]. These and other limitations, as well as adapted solutions, have been discussed in [14,15]. Sammon's method may include several data overlaps when the *n*-dimensional input space contains noisy or weakly discriminatory information [30]. Depending of the size of the input data (number of points), the number of learning iterations and computational facilities available, Sammon's mapping might be computationally expensive. Empirical analyses have shown that Sammon's mapping may easily get stuck at local optima [30]. Moreover, it has been demonstrated that this method may be sensitive to the initialisation scheme applied [19,31].

This paper assesses an alternative, non-linear mapping technique which aims to address key limitations exhibited by traditional methods. Its application to clustering-driven exploratory analysis of gene expression data is investigated. Furthermore, it provides the basis for an interactome network clustering visualisation system. The following section summarises relevant results.

## Results

**Figure 1:**
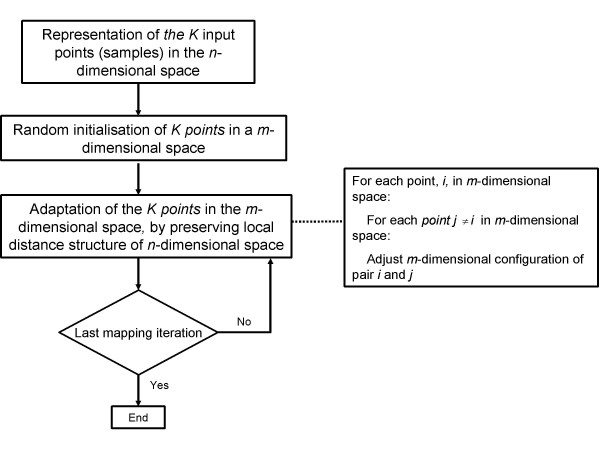
**Relaxation method for non-linear mapping. **The input to the algorithm is a collection of *K *points described in an *n*-dimensional space. A mapping iteration refers to a complete sequence of adaptation steps involving pairs of points, *p*(*i*,*j*). A point may encode a biological sample described by a gene expression profile (e.g. tumour sample), or a protein described by an interaction profile.

A relaxation method for non-linear mapping was implemented to visualise relevant similarity relationships in data originating from gene expression and interactome data. Such a method was designed by Chang and Lee [32] to address key limitations observed in methods such as those proposed by Sammon [25] and Kruskal [23,24]. These techniques are related because they aim to achieve a space reduction by preserving the structure of local distances in the data. However, unlike those traditional mapping techniques, the method assessed in this paper adapts a pair of points in the transformed *m*-dimensional space at every processing step, instead of adapting all points at every step. Thus, the term "relaxation" is taken from the relaxation method for linear equalities [32]. Chang's and Lee's method showed to outperform Sammon's mapping both in terms of cluster detection effectiveness and computational efficiency. A *mapping iteration *is defined as a complete sequence of adaptation steps involving pairs of points, *p*(*i*,*j*), for each *i *≠ *j *(see Methods for a more detailed description). In this study a point may encode a biological sample described by a gene expression profile (e.g. tumour sample), or a protein described by its interaction profile. Figure 1 summarises the mapping mechanism of this approach.

### Analysis of gene expression data

Analyses were performed on three publicly available expression data sets. The first one includes 38 samples from a known leukaemia study [33], which are represented by 50 expression values. The samples are categorised into two classes: *Acute myeloid leukemia *(AML) and *acute lymphoblastic leukemia *(ALL). This data set has been previously validated by several experimental and *in silico *methods. It may be considered as an adequate example for illustrating basic capabilities of clustering algorithms. The second data set includes samples originating from *small, round blue-cell tumours *(SRBCT) [28]. These data consisted of 63 samples categorised into four classes: *Ewing family of tumors *(EWS), *rhabdomyosarcoma *(RMS), *Burkitt lymphomas *(BL) and *neuroblastomas *(NB), which are represented by the expression values of 2308 genes with suspected roles in processes relevant to these tumours. The third data set offers another example of gene expression analysis complexity: A data set consisting of a relatively small number of samples described by thousands of gene expression values, without incorporating pre-processing by feature selection or transformation procedures. This application comprises 20 samples described by 9504 gene expression values for both *normal brain *and a pharmacological model of *Parkinson's disease *[34]. The reader is referred to the section of Methods for a more detailed description of the data and their prediction tasks.

Several experiments were performed on each data set to assess possible, key advantages and limitations of the mapping approach introduced above. Each experiment requires an input file storing a matrix, **A**, where each entry, *a*_*ij*_, represents an expression value, *j*, for a sample, *i*. The user needs to define only one learning parameter, the number of mapping iterations, as defined above. The output of the algorithm is the projection of the *K *samples represented in **A **on the reduced *m*-dimensional space. Mappings were analysed for *m *= 2 and *m *= 3, which allowed the generation of 2D and 3D visual displays. Experiments were also conducted for several numbers of mapping iterations. The results facilitated a high-level understanding of fundamental similarity relationships between the samples, which are also consistent with previous research. Three important exploratory data analysis tasks were accomplished: The automated, unsupervised detection of clusters relevant to the natural class structure of the data sets; the visualisation of a coherent preservation of local similarity (distance) relationships between samples; and the identification of potential outliers.

**Figure 2:**
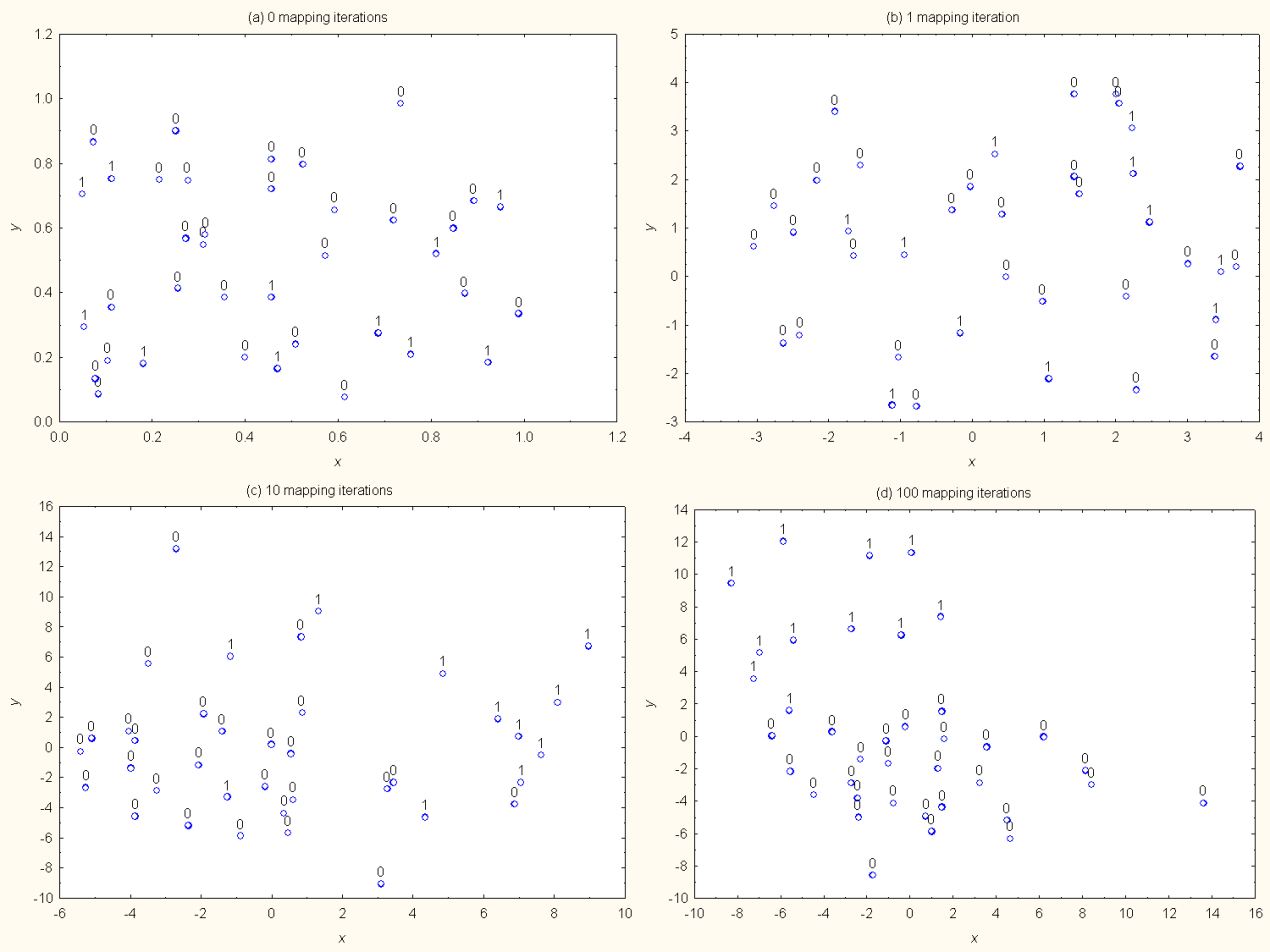
**Visualisation of clusters in leukaemia data for different numbers of mapping iterations. **Panels (a) to (d) show results for 0, 1, 10 and 100 mapping iterations respectively. Circles are used to represent the samples on the maps. Labels '0' and '1' refer to ALL and AML samples respectively.

Figure 2 depicts mapping results for the leukaemia data. Panels (a) to (d) show results for 0, 1, 10 and 100 mapping iterations respectively. Circles are used to represent the samples. Labels '0' and '1' refer to ALL and AML samples respectively. In the initialisation of the mapping process (0 iterations) the samples are randomly assigned to positions in the 2D space. After 10 iterations there is a clear indication of separation of samples belonging to different classes. With 100 iterations the ALL samples are clustered on the upper left side of the map, and the AML samples are clustered at the bottom of this map. Such clusters were also distinguished in different experiments. As a consequence of the random initialisation process, clusters may occupy different areas on the resulting maps for different experiments using the same number of iterations. Nevertheless, the system correctly separated samples in different experiments with more than 20 iterations.

**Figure 3:**
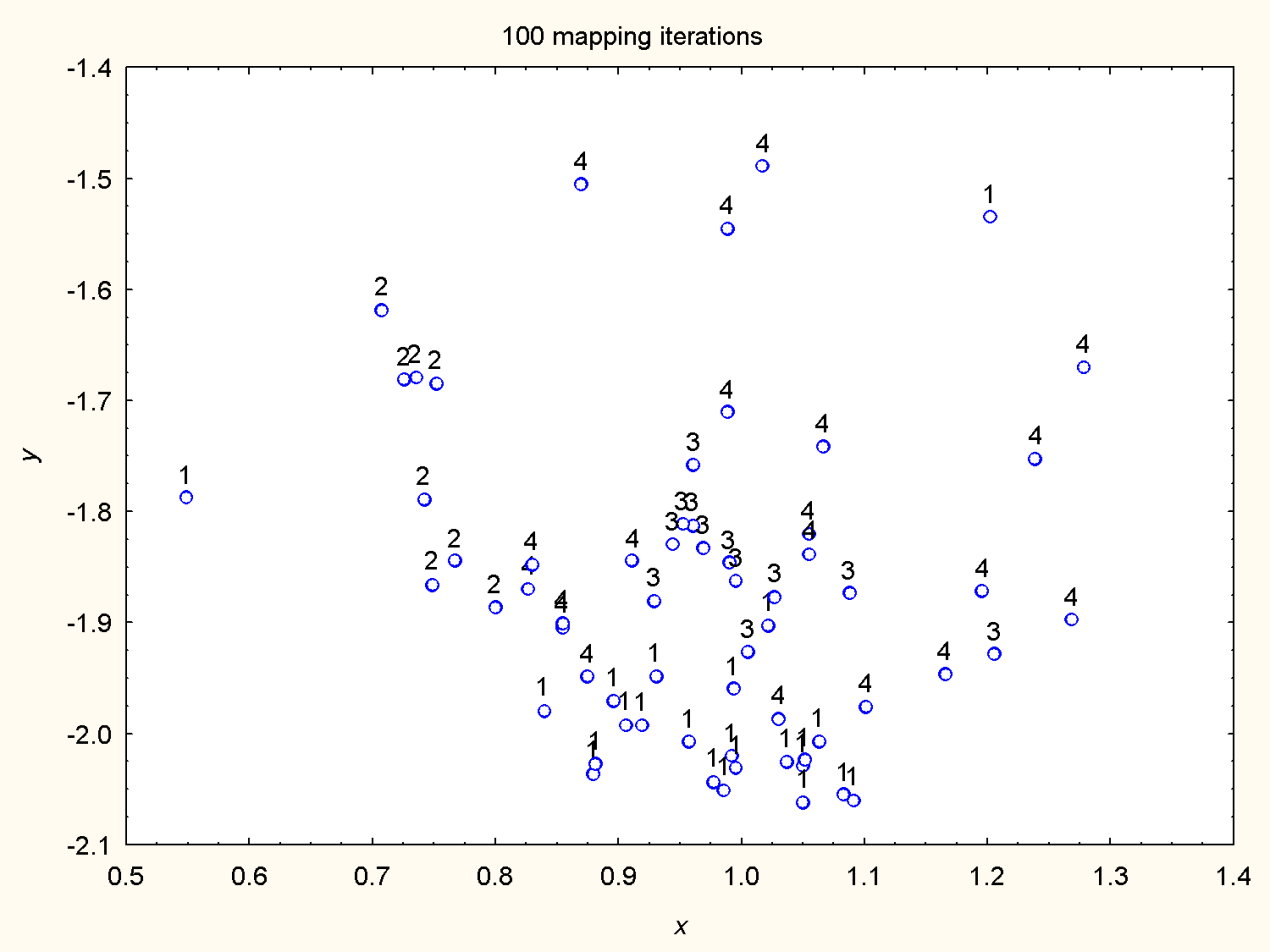
**Resulting maps for the SRBCT data. **EWS, RMS, BL and NB samples are represented by symbols '1', '2', '3' and '4' respectively. 100 mapping iterations.

Figure 3 shows resulting maps for the SRBCT data with 100 mapping iterations. These data were pre-processed as explained in the section of Methods. EWS, RMS, BL and NB samples are represented by symbols '1', '2', '3' and '4' respectively. It suggests that the mapping process was able to identify key similarity relationships. Samples belonging to the same class tend to cluster together. For example, EWS samples are mainly located at the bottom of the map. RMS samples are clustered on its left side. The sample '1' (*x*: 0.54, *y*: -1.79) that was located on the left side of Figure 3 far from the EWS cluster (its natural class) was consistently mapped in this fashion by different experiments. Moreover, this sample was also displayed closer to RMS samples for different experiments, which might suggest a significant relationship between such a sample and the RMS class. Furthermore, a previous study using more sophisticated, supervised learning models showed that this sample may be difficult to correctly classify [35]. This suggests that the map shown in Figure 3 highlights a potential outlier in the EWS class data.

**Figure 4:**
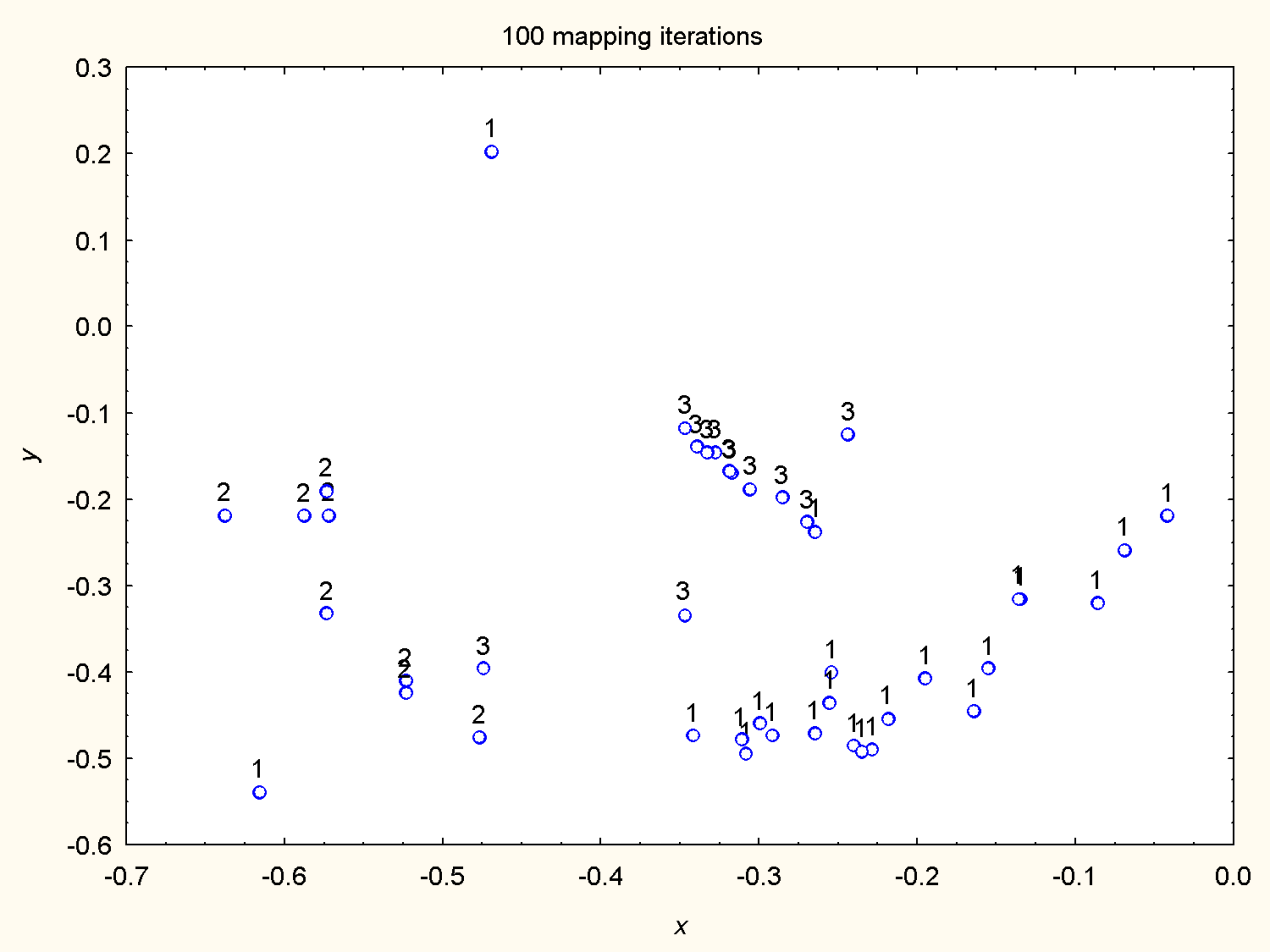
**Resulting maps for the SRBCT data considering only 3 classes. **EWS, RMS and BL samples are represented by symbols '1', '2', '3' respectively. 100 mapping iterations.

Additional experiments using a smaller number of SRBCT classes (only EWS, RMS, BL) were performed. This was mainly done to explore the possibility of obtaining alternative graphical views of the data. Results are in general consistent with the results produced with 4 classes: Samples belonging to the same class were clustered together. However, these experiments allow a clearer graphical differentiation of classes on the resulting maps. Figure 4 depicts an example obtained with 100 mapping iterations. For this and other experiments, it was also possible to detect the outlier that was observed in Figure 3. Such an EWS sample is located at the top of the map shown in Figure 4.

**Figure 5:**
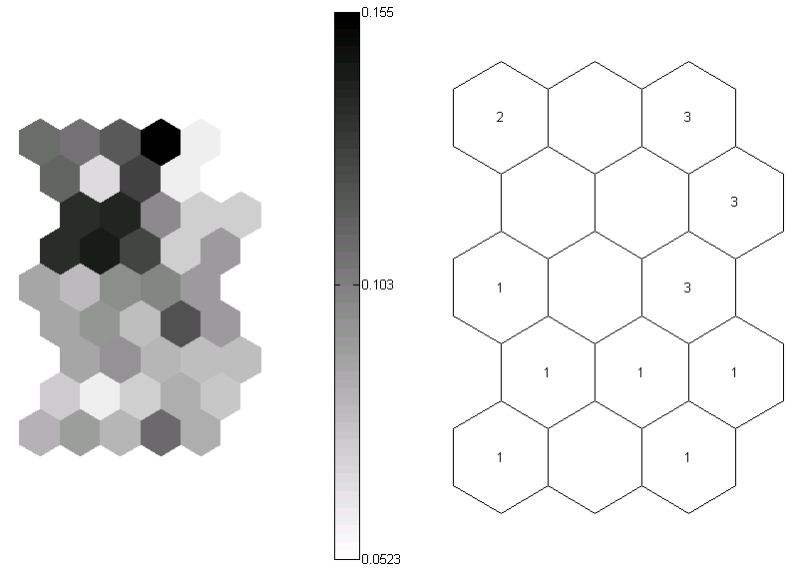
**SOM-based analyses on the SRBCT data. **U-matrix and label map (panel on the right side). U-matrix depicts the distances between neighbouring map units using a grey scale. In the label map a node represents a class based on a majority voting strategy for the samples associated with this node. EWS, RMS and BL samples are represented by symbols '1', '2', '3' respectively. Additional file 1 shows its frequency map.

SOM-based analyses using the *SOM Toolbox *[36,37] were also implemented to establish comparisons. Figure 5 shows the *unified distance matrix *(*U-matrix*) and the *label map *(panel on the right side) for a representative result. The section of Methods describes the construction of these maps. The SOM was able to correctly cluster the SRBCT samples. However, these standard SOM visualisation techniques do not provide clear information on sample-to-sample similarity relationships. Moreover, they do not adequately facilitate a direct visualisation of the distribution of samples assigned to each node and their associations. Additional file 1 includes the frequency map, which is another standard SOM visualisation technique, for these results.

**Figure 6:**
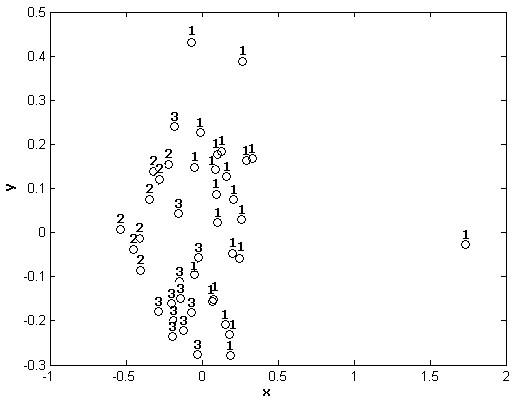
**Sammon's mapping analyses on the SRBCT data. **Symbols "1", "2" and "3" represent classes EWS, RMS and BL respectively.

Figure 6 shows results obtained by applying Sammon's mapping. Symbols '1', '2' and '3' represent classes EWS, RMS and BL respectively. This approach is able to detect class differences between samples. Sammon's mapping also isolated the EWS sample suggested above as a possible class outlier (right side of the map). Figure 3 (near *x*: 1.2, *y*: -1.5) suggests another sample '1' as a potential outlier. However, the Sammon's mapping did not clearly depict it as a potential outlier because in this case, unlike Figure 3, this sample is located closer to class '1' samples (Figure 6, near *x*: 0, *y*: 0.4).

3D Mapping analyses were also implemented for this SRBCT application. Additional files 2 and 3 compare the results originating from the relaxation non-linear and Sammon's mapping methods. Both methods displayed coherent partitions of the data, which are consistent with the 2D mapping results presented above. Moreover, 3D mappings also suggest relatively strong similarities between EWS and RMS samples because of their proximity on the maps.

**Figure 7:**
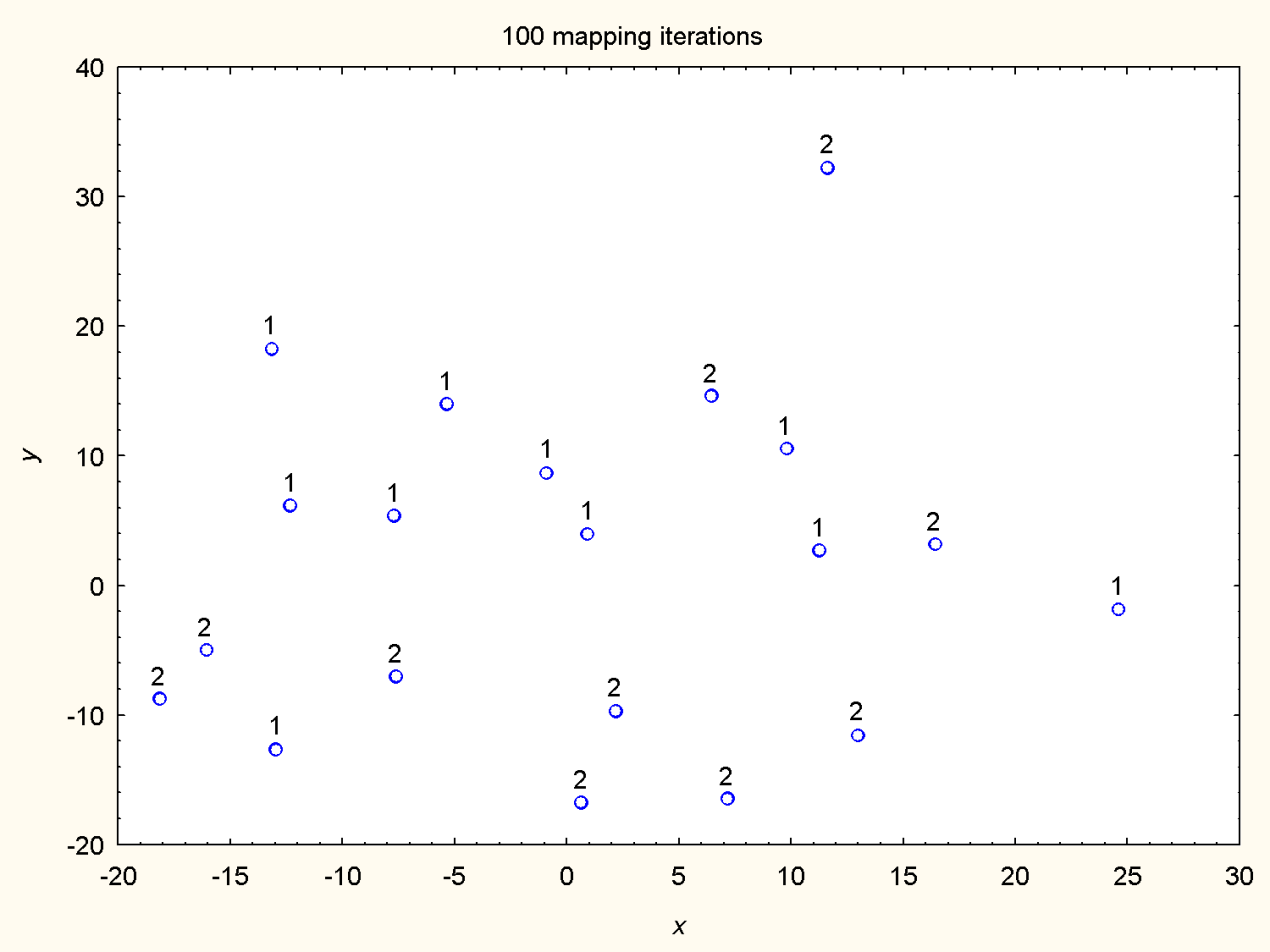
**Relaxation non-linear mapping of the Parkinson's disease model data. **Parkinson's disease and Normal samples are identified by symbols '1' and '2' respectively. 100 mapping iterations.

**Figure 8:**
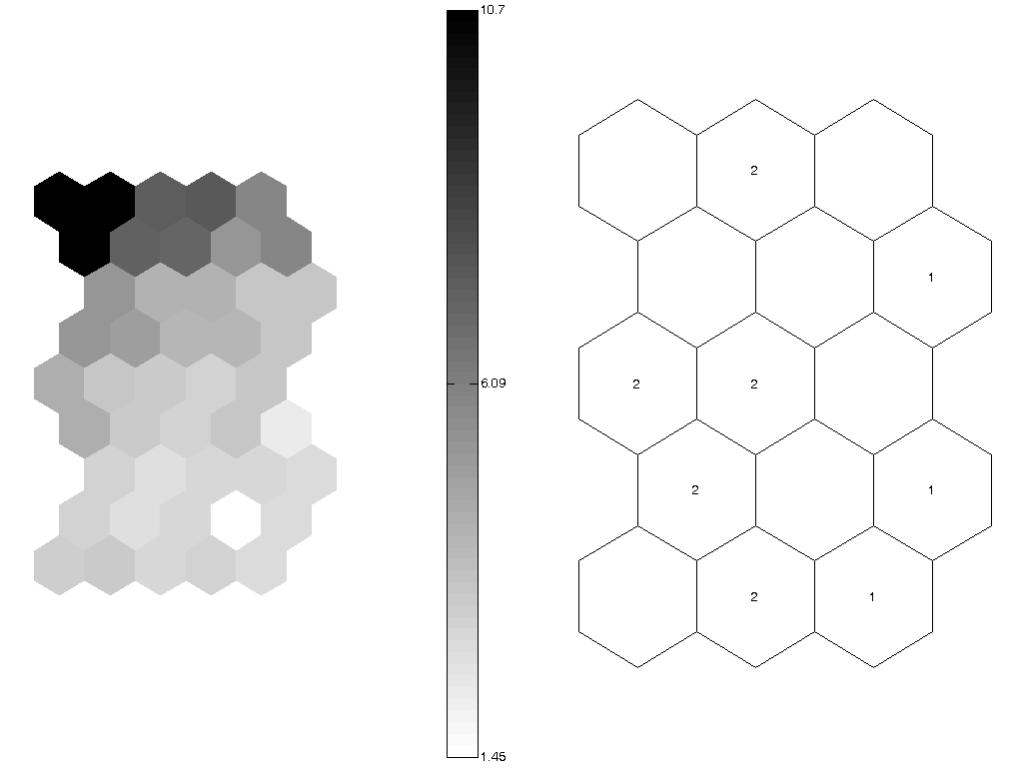
**SOM of the Parkinson's disease model data. **U-matrix and label map (panel on the right side). U-matrix depicts the distances between neighbouring map units using a grey scale. In the label map a node represents a class based on a majority voting strategy for the samples associated with this node. Additional file 4 shows its frequency map.

**Figure 9:**
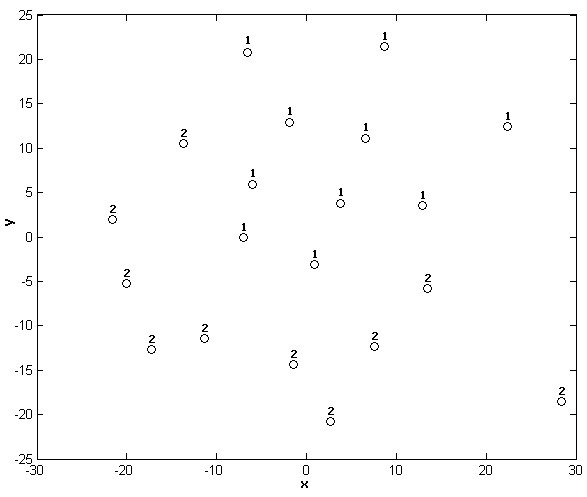
**Sammon's mapping of the Parkinson's disease model data. **Symbols "1" and "2" represent Parkinson's disease and Normal samples respectively.

Figure 7 displays a relaxation non-linear map of the Parkinson's disease model data. Parkinson's disease and Normal samples are identified by symbols '1' and '2' respectively. After 100 mapping iterations, results suggest that the method is able to differentiate between these classes. Parkinson's disease samples mainly fall on the upper region of the map (*y *> 0), and 7 (out of 10) Normal samples are located below that area. Figure 8 shows SOM-based results, which adequately distinguish between classes. It also indicates that a few Normal samples may be closer to Parkinson's samples than to the main group of its own class. Figure 8 may offer a clearer graphical discrimination between classes. However, the SOM-based results are more dependent on the user's selection of an optimum set of learning parameters (see Methods). Additional file 4 shows the frequency map for these results. Although the separation of clusters is less clear, Sammon's mapping (Figure 9) was in general capable of grouping same class samples. In this figure symbols '1' and '2' represent Parkinson's disease and Normal samples respectively. 3D maps originating from relaxation non-linear and Sammon's mapping methods are depicted in Additional files 5 and 6 respectively. Both techniques offer alternative, but consistent exploratory views of the cluster structure of this data set.

### Analysis of interactome networks

As a way of promoting alternative, potential applications of the methodology presented, this section illustrates an example of exploratory data analysis of interactome networks. The approach proposed encodes a network as a graph of interconnected nodes. For a network consisting of *N *nodes, the mapping tool (from now on referred to as *interClust*) requires an *N *× *N *matrix, **B**, as the input data. In this symmetrical matrix each element, *b*_*ij*_, represents the *connection strength *between nodes *i *and *j *in the graph. Such values may also be interpreted as *weights *representing the relevance of the interaction between a pair of proteins, e.g.: number of hits observed in interaction experiments or a path distance between two nodes on the graph (indirect interactions). Thus, each row in **B **may be seen as the *connectivity profile *for a node, *i*. That is, the connectivity profile of a node becomes this node's coordinates in the *n*-dimensional, input space. An accompanying tool, *inBuilder*, automatically generates such a network representation from a list of pairwise protein-protein interactions and their respective connection strengths predefined by the user. The section of Methods provides more information about design and operation aspects of this approach.

**Figure 10:**
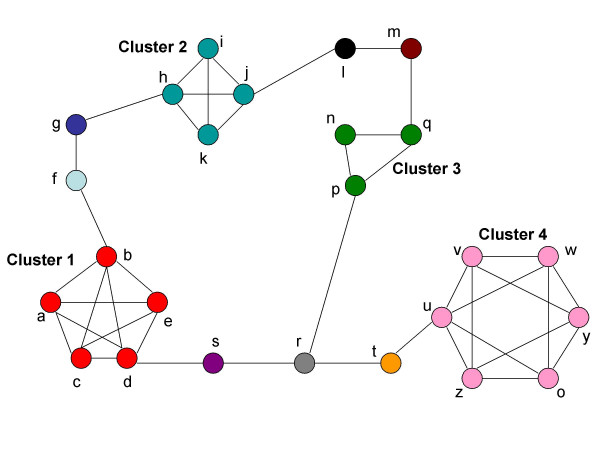
**Example of a network characterized by a number of clusters. **The length of the links does not reflect distances or connection strengths. All of the direct connections are considered equally.

**Figure 11:**
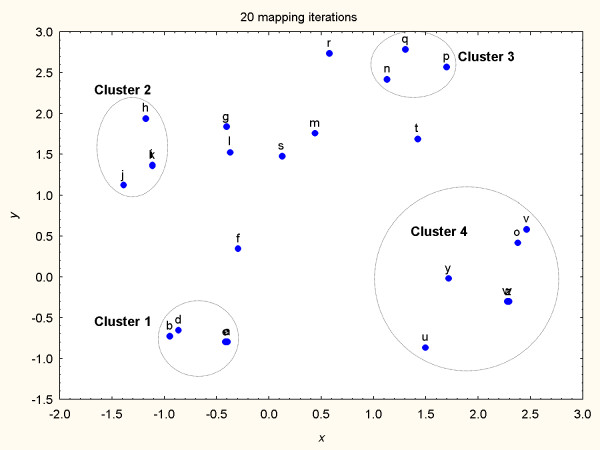
**A resulting map after applying interClust to the network shown in Figure 10. **Clusters are identified and local interaction relationships tend to be preserved. 20 mapping iterations.

Before testing this approach on a real interactome data set, a simple example of a network (Figure 10) is used to illustrate its application. In this figure the length of the links does not reflect distances or connection strengths. All of the direct connections are considered equally. This network comprises 25 nodes forming 4 main clusters. In this case a cluster is defined as a compact group of interconnected nodes. Figure 11 shows the results obtained by applying *interClust *to this network with 20 iterations. This map clearly distinguishes the clusters of the network. Moreover, it preserves local interaction relationships: i.e. if two nodes have similar connectivity profiles in the original space (Figure 10), then they are also close to each other in the resulting map. Adequate cluster visualisations were also obtained in experiments with more than 5 iterations.

**Figure 12:**
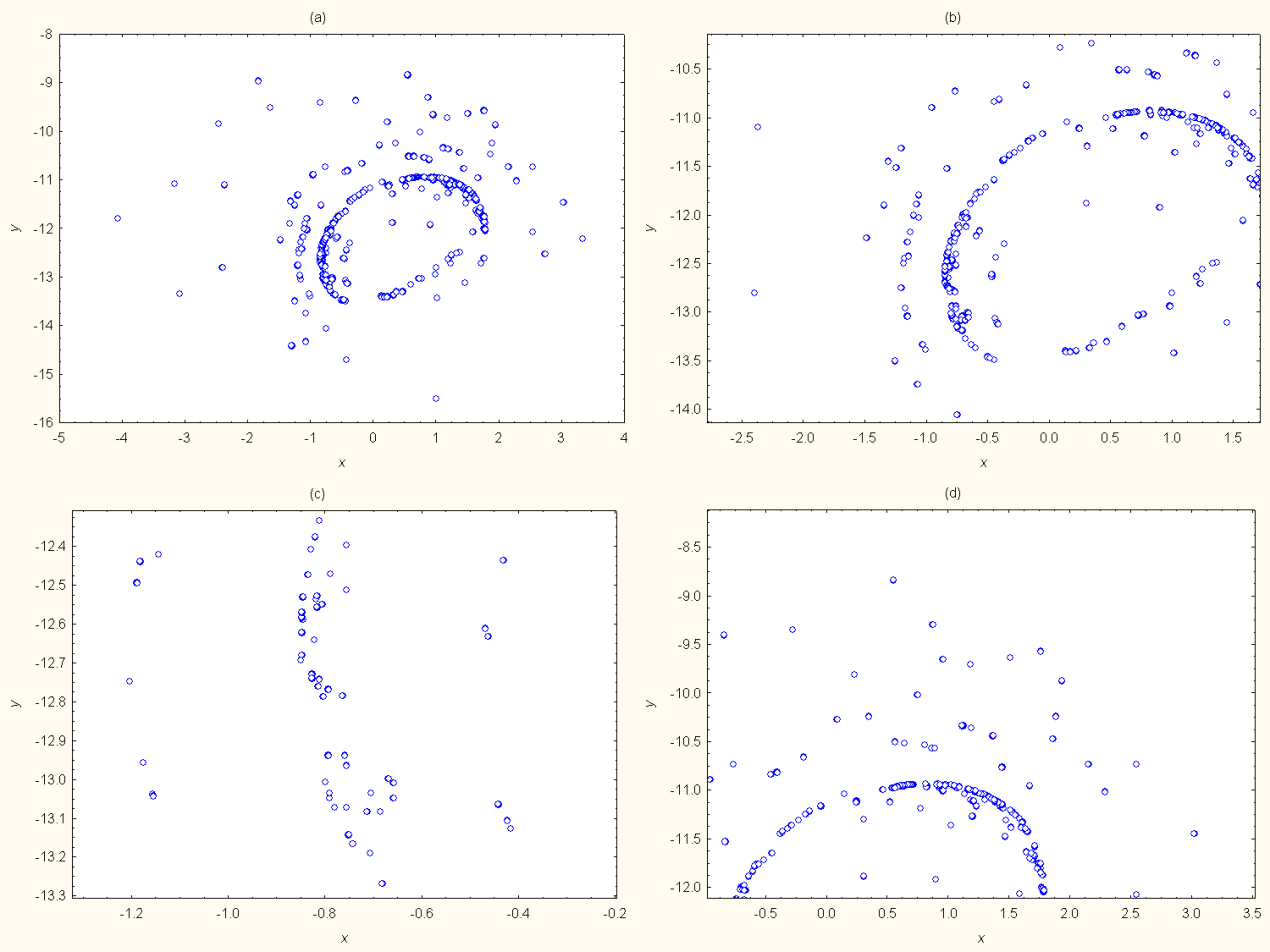
**Relaxation map for the chromatin interactome network. **Each panel depicts different regions and levels of detail, obtained with 100 iterations.

This algorithm was tested on the chromatin interactome in *C. elegans*. It includes 303 proteins and 349 interactions. This data set regroups interactions of proteins involved in transcriptional regulation at the chromatin level. This functional module is of major interest because it is at the crossroads of many biological processes such as development, sex determination, cellular differentiation and proliferation. Its misregulation may have strong consequences such as tumorigenesis or developmental defects [38]. This data set includes both retested [39] and non-retested protein interactions obtained by high-throughput two-hybrid screens (unpublished data). Figure 12 displays resulting relaxation maps, at different regions and levels of detail, obtained with 100 iterations.

**Figure 13:**
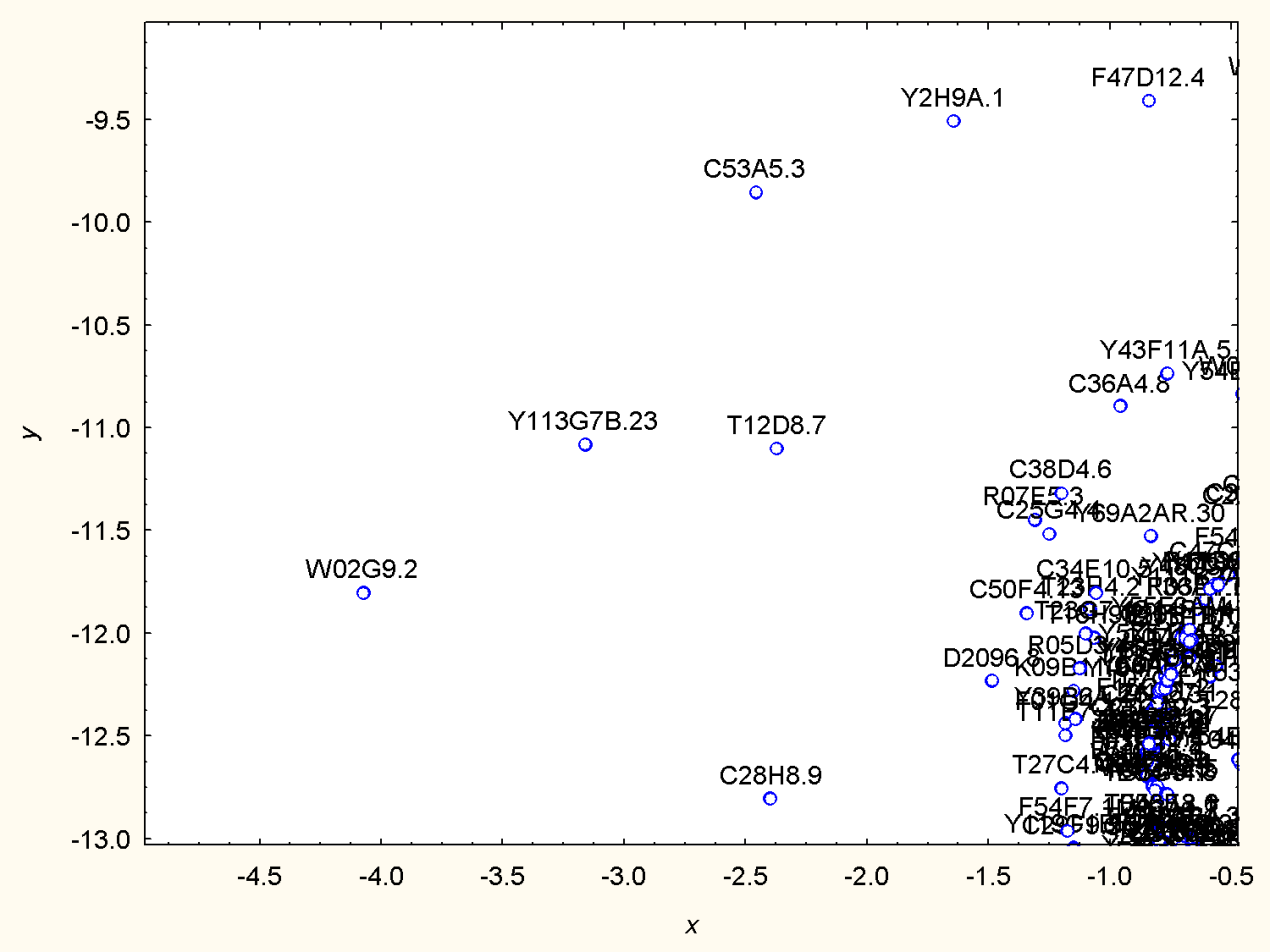
**Partial view of chromatin interactome map. **Its shows a region enriched by hubs, which is well-separated from the main group of proteins.

Figure 12 indicates a separation of proteins according to their connectivity patterns. The network encoding scheme and the non-linear mapping algorithm applied distinguished highly-connected proteins (*hubs*) from the other components of the network. Hubs are located in the outer regions of the map. The farther a node is from the centre, the more connections it has. Figure 13 displays a partial view of a region enriched by hubs, which is well-separated from the main group of proteins. The hubs are also relatively well-separated between them. It reflects the fact that such proteins share very few direct, interacting proteins in common.

Additional file 7 depicts some of the hubs automatically isolated by the mapping algorithm, as well as a few nodes located near the centre of the map (F15A2.6, C34E10.5, C14B9.6). These diagrams were drawn using the *InterViewer *tool [40]. A closer examination of a group of proteins located in the outer regions of the map (Figures 12 and 13), for example, reveals that they are involved in key processes such as mitosis and meiosis. This differentiation indicates that such hubs may act as connection components between different biological processes. This is not a surprising finding, but it highlights the capacity of the algorithm to automatically detect some of the most biologically-relevant proteins only on the basis of their connectivity profiles. Seven of these hubs (Y2H9A.1, F56C9.1, F11A10.2, T12D8.7, Y113G7B.23, Y37D8A.9, C53A5.3), for instance, show a significant enrichment of phenotypes obtained by depletion of the transcripts by RNA interference (5.6-fold enrichment compared to genome-wide levels [41]). Moreover, four of these hubs (F56C9.1, F11A10.2, T12D8.7, C53A5.3) are strongly linked to embryonic lethality (*Emb*) by exhibiting a 6-fold enrichment of this property in relation to genome-wide levels [41].

**Figure 14:**
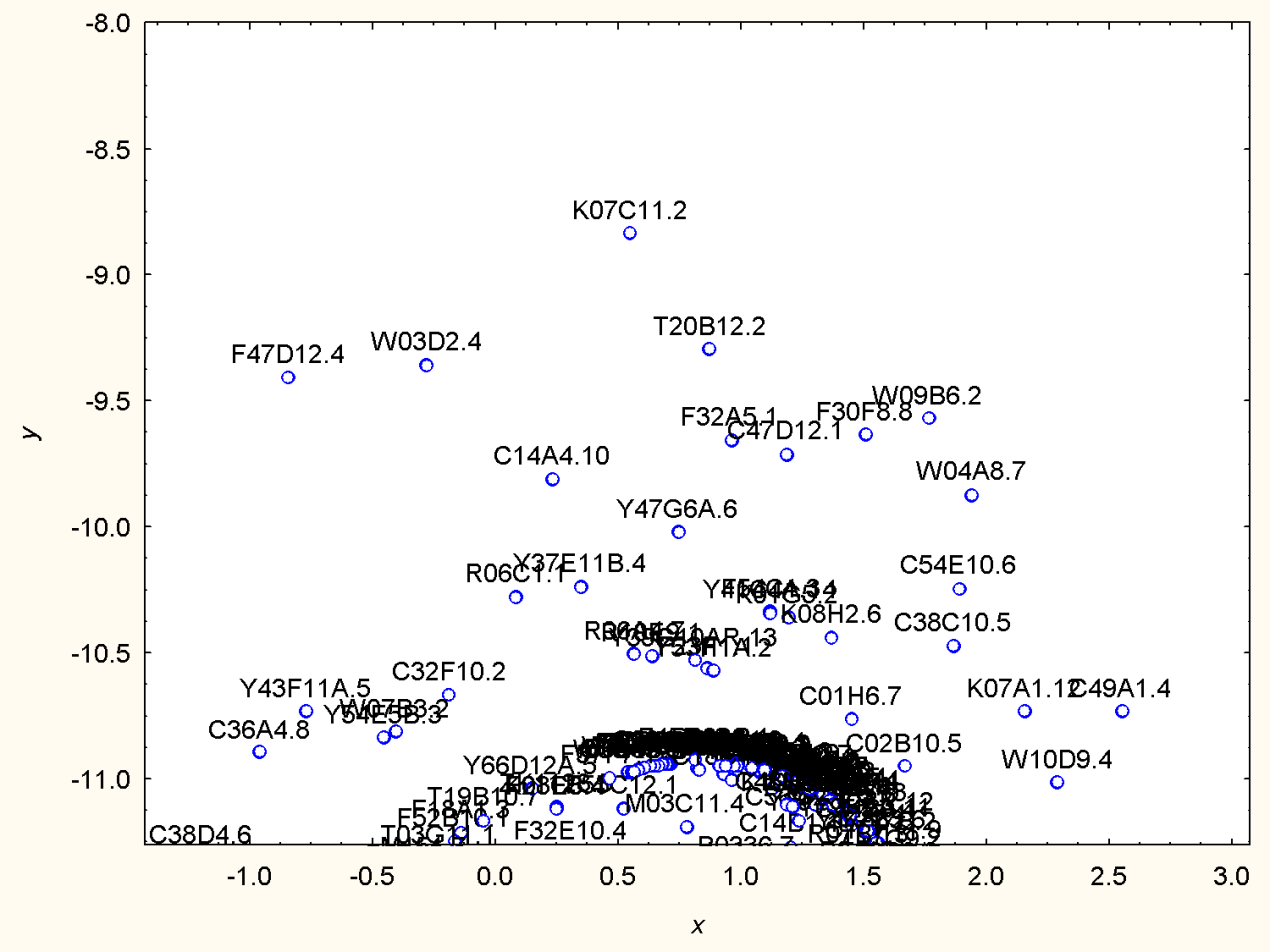
**Identification of key functional components based on cluster visualisation. **The region surrounding protein T20B12.2 includes several components of the core polymerase II enzyme, as well as several related families that share similar phenotypes. See Additional file 8 for further descriptions.

Further analyses on the map suggest that neighboring nodes may reflect functional similarity relationships between the corresponding proteins. One example is illustrated in Figure 14 (upper area), which focuses on the region surrounding T20B12.2, also known as Tbp-1, a key component of the Polymerase II holoenzyme. In this region it is possible to identify several components of the core Polymerase II enzyme, as well as several related families (histones acetylases and deacetylases, nucleosomes positioning enzymes of the Swi/snf family) that share similar phenotypes. There is a strong enrichment of phenotypes such as embryonic lethality or problems in growth. These phenotypes (like embryonic lethality) are often attributed to proteins with high connectivity. Only eleven (out of twenty seven) proteins examined in this region exhibit wild type phenotype when they are depleted. This cluster shows a 5.4-fold enrichment of phenotypes, a 5.1-fold enrichment of Emb phenotype, and a 33.3-fold enrichment of sterile progeny phenotype in comparison to genome-wide levels [41]. This cluster also exhibits significant associations with sterility, growth defect and problems in larval development. Additional file 8 describes the composition of this representative cluster.

## Discussion

With regard to gene expression data, the relaxation non-linear mapping method was capable to support an automated, unsupervised detection of relevant clusters of samples. Results demonstrated that it may also be useful for the visualisation of local similarity relationships between samples and the identification of potential outliers. In general its performance was comparable to Sammon's mapping. One cannot of course expect that a single method would always be able to accurately map different types of high-dimensional data. However, the application of both techniques is recommended as a reliable approach to data exploratory analysis. In this study they offered consistent views of the problems under analysis. SOM, as well as many other cluster analysis techniques, may be more suitable for application after first gaining an adequate insight into the structure and organisation of the data. Such a global understanding may be facilitated through the application of different non-linear mapping methods.

Even though a comparison with existing network clustering methods was not implemented, preliminary results suggest that the approach proposed might represent a useful tool for interactome network visualisation and clustering. It distinguished key hubs and facilitated the identification of functionally relevant clusters. It showed that, not surprisingly, most of the hubs detected are essential for the normal development, behaviour and reproduction of *C. elegans *by exhibiting an enrichment of phenotypes obtained from RNA interference. This can be explained by the fact that the partners connected to these hubs are involved in numerous fundamental processes such as mitosis or meiosis. Hence, the modification of the network caused by the absence of a hub may have strong consequences on the topology of the network and the organisation of the cellular processes. Isolation of functional clusters (e.g. chromosome condensation and segregation of the transcriptional core process) is essential to investigate relationships between groups of proteins or modules. Modules playing a role in the same process (e.g. chromosome condensation and segregation during the mitosis) also tended to be interrelated in the clustering analysis. This underlies the fact that these modules are also functionally interconnected and are interdependent to stringently regulate the cellular processes. Without these connections the process may be misregulated and generate aberrant behaviour (i.e. oncogenesis if the mitosis is not well regulated). In this way, interactions that connect these modules are likely to be at the intersection of several biological processes and to regulate the correct succession of events in a cellular process (e.g. condensation of chromosomes before segregation). We do not claim that the tool reported can be used as an interaction prediction technique. The example analysed aims to illustrate the application of exploratory data analysis for detecting regions, which may be biologically meaningful and relevant for assessing the outcomes from protein-protein interaction prediction techniques. Such patterns may be useful for guiding future computational or experimental analyses to validate interaction hypotheses. Meaningful patterns may be associated, for example, with functionally enriched regions, as shown in this paper. Moreover, because of the limitations regarding predictive accuracy and coverage exhibited by existing single-source techniques, it is important to offer user-friendly tools that may help scientists to detect possible, spurious associations. Future research should include a more exhaustive statistical description of all possible hub candidates detected by the mapping process for this and other network examples. Statistical and functional attributes represented by this method should be compared with previous findings from large-scale, comprehensive studies. Such an investigation was not implemented here because it is outside the main scope of this paper.

The preservation of local distance structures is an important property to interpret the non-linear mapping techniques studied here. This is the main goal of their data transformation mechanisms. It basically means that the distance between two points, *dm*_*ij*_, in the transformed *m*-dimensional space should be very similar to *dn*_*ij *_(their distance in the original *n*-dimensional data) if *dn*_*ij *_is small. However, if *dn*_*ij *_is relatively large, *dm*_*ij *_is not required to be similar to *dn*_*ij*_. A fundamental difference between the relaxation non-linear and the original Sammon's mapping methods is that the former adapts a point-to-point distance at every processing step, instead of adapting all of the distances at every step.

For relatively small data sets the computing times required by the Java-based implementation of the relaxation non-linear mapping method were comparable to those obtained from *Matlab*^® ^implementations of SOM and Sammon's techniques, i.e. in the order of seconds. But for larger data sets, e.g. Parkinson's disease data, the non-linear mapping method may run in the order of minutes. This of course depends on the number of mapping iterations and memory resources available. This concern may be addressed by implementing an optimised version of the software, perhaps using another programming language. Another important solution is the implementation of a *frame method *[32], which has demonstrated to improve the computational efficiency of the algorithm without significantly compromising its data structure preservation capabilities. We also intend to expand this and related research within an open-source data analysis and visualisation platform, such as the *TIGR Multiexperiment Viewer *[42].

An important aspect of future research is the adaptation of the relaxation non-linear mapping method to perform tasks beyond exploratory data analysis. A desirable property would be its capacity to generalise solutions for new samples in an incremental fashion. That is, the system should be able to add new samples to a map without having to re-generate it. One possible solution is the application of an artificial neural network to interpolate and extrapolate the mapping as illustrated by Mao and Jain [43]. It is also important to develop hybrid systems to combine the strengths and advantages demonstrated by various mapping techniques [44]. A two- stage approach, for example, represents a feasible solution. In this approach a data set may be firstly partitioned into a set of *Voronoi *spaces using clustering techniques such as *k*-means and SOM, and then independent mapping projections may be performed on each area. It has been suggested that such a hybrid model may be advantageous especially when dealing with massive data sets [45]. Other investigations will involve the assessment and comparison of related techniques [46,47]

The mapping algorithm successfully recognised key topological properties and functional relationships in an interactome network based on a graph encoding scheme, which only considers direct interactions. Moreover, it considered all graph connections as equals. Nevertheless, it would be important to implement applications in which the network encoding values, *b*_*ij*_, may also reflect non-direct, shared interactions. This may be done, for example, by defining a graph distance function between network nodes. Another input representation scheme may exploit information relevant to the significance or confidence assigned to the interactions based on experimental evidence.

Since cellular networks are organized in a modular fashion, the identification of these modules is crucial to understand relationships between biological processes and offer a higher-order, more accessible representation of the interactomes. The clustering approach proposed in this paper provides a meaningful, simplified representation of complex interactomes. This representation may significantly facilitate exploratory analysis of networks for non-specialists in bioinformatics. This type of analysis is fundamental to detect key network components, such as hubs, which are implicated in many physiological disorders. Identifying these hubs and their associated clusters is also an important step toward the functional annotation of these proteins, as well as for obtaining possible explanations of their involvement in a specific disease. The cluster visualisation tool, *interClust*, may represent a useful technique to analyse other protein-protein interaction networks including a future human interactome. For instance, it may be applied to isolate proteins linked to a human pathology and to associate them with a cluster or functional modules (e.g. the transcriptional core complex of the Polymerase II enzyme). Another important component of future research is the adaptation of network clustering methods to take into account spatio-temporal aspects of interactions based on, for example, microarrays, in-situ hybridization or protein localization data. Non-linear mapping methods may also be applied to support the annotation of unknown proteins. This may be done by assigning a protein to a functional role that is significantly associated with the cluster under consideration. Furthermore, it is of course fundamental to compare this network clustering methodology with existing techniques. Thus, such approaches may aid researchers in the design of further experiments and the selection of more sophisticated bioinformatics analyses.

## Conclusions

This research studied a user-friendly cluster visualisation approach that is able to support the generation of biologically meaningful outcomes. It represents an effective and robust exploratory data analysis technique. Comparisons indicate that applying more than one mapping approach may improve the confidence of results. Moreover, this may facilitate the generation of alternative, meaningful views of the data. Relaxation non-linear and Sammon's mapping techniques may be more suitable for exploratory data analysis tasks than SOM.

This study did not aim to add another algorithm to the existing collection of supervised and unsupervised classification tools. This methodology is not reported as a competing solution to clustering algorithms. Our study shows how an exploratory data analysis approach based on non-linear mapping can support the identification of relevant, biologically-meaningful patterns. We do not argue that the methodology proposed should necessarily offer more accurate results in relation to existing classification solutions. We recommend this methodology as a first step towards understanding complex data mining problems in functional genomics. Such an exploratory approach may also facilitate the selection of more sophisticated methods and highlight possible, critical features for successfully implementing clustering-based studies. This research indicates that the outcomes originating from an exploratory, pattern visualisation method may be as meaningful as those produced by more sophisticated classification approaches, i.e. SOM. Moreover, the methodology proposed does not require the user to define multiple learning parameters.

Exploratory analysis frameworks may facilitate a better insight into a data set before applying more sophisticated, problem-specific classification or predictive models. Such an insight may be achieved by helping users to recognise key features of the underlying structure of the data, detect potential outliers or anomalies and test assumptions about the cluster composition of the data.

An adaptation of the relaxation non-linear mapping technique, *interClust*, represents a promising solution to aid researchers to recognise key connectivity and functional patterns in interactome networks. Further research is underway to continue assessing its application to this area.

## Methods

### Data

The leukaemia data set includes 38 samples originating from [33]. Each sample is represented by 50 expression values. The samples are categorised into two classes: *Acute myeloid leukemia *(AML) and *acute lymphoblastic leukemia *(ALL). The original data sets and experimental protocols can be found at the Broad Institute Web site [48]. For each feature standardisation was applied by subtracting each value from the mean and dividing it by the standard deviation.

The SRBCT data consisted of 63 samples categorised into four classes: *Ewing family of tumors *(EWS), *rhabdomyosarcoma *(RMS), *Burkitt lymphomas *(BL) and *neuroblastomas *(NB), which were represented by the expression values of 2308 genes. The dimensionality of the SRBCT expression samples was reduced by applying PCA. It has been shown that the application of PCA is important to facilitate an adequate discrimination of samples in this data set. The 10 dominant PCA components for each case were used as the input to the analysis techniques as suggested by [28], who applied a supervised learning approach to classify the samples after reduction by PCA. Using the raw data (without PCA) the relaxation non-linear mapping was not able to adequately depict differences between the samples. The original data sets and experimental protocols can be found at the National Human Genome Research Institute Web site [49].

The Parkinson's disease data include 20 samples described by 9504 gene expression values for both *normal brain *(10 samples) and a pharmacological model of *Parkinson's disease *[34]. *M. musculus *was the organism studied in this disease model. The data are available at The Gene Expression Omnibus [50] (accession number GDS22).

Additional files 9 to 11 include, respectively, the leukaemia, SRBCT and Parkinson's disease data analysed in this paper.

The network of interactions was derived from the chromatin interactome in *C. elegans*. It contains 303 proteins and 349 interactions. It comprises components of the Polymerase II holoenzyme, histones modifying enzymes, nucleosomes positioning proteins and several proteins containing domains known to be essential to this process such as the chromodomain, the bromodomain or the SET domain. This is an early version of the chromatin interactome, which includes a number of retested [39] as well as non-retested interactions determined by a stringent high-throughput two-hybrid screen. It also contains several interologs [38,51]. A combination of experimental and bioinformatic factors (reporter genes used for the phenotypic tests, number of hits per interactions, a blast e-value less than 1E-10, a PHRED score >20 for 15% of the ISTs (interaction sequence tags) and the frame verification method) were used to provide optimal accuracy. It is known that the two-hybrid approach has the tendency to generate more false positives than the pull-down/Mass spectrometry approach, for example. However, in the data set analysed the rate of false positives is reduced by using more reporter genes (up to 4 genes, unlike traditional large scale two-hybrid screens which commonly use 2 genes). Using 4 reporter genes can reduce the rate of false positives up to 50%. Additional file 12 contains this data set.

### Algorithms and tools

The relaxation non-linear mapping algorithm is summarised in Figure 1 and details on its design are reported in [32]. The adaptation of a pair points, *i *and *j*, in the transformed, *m*-dimensional map is implemented as follows. Given two points, *Pm*_*i *_and *Pm*_*j*_, in the *m*-dimensional map, the adjusted new values, *Pm*_*new*, *i *_and *Pm*_*new*_, _*j*_, are calculated using:


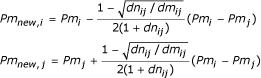


where *dn*_*ij *_and *dm*_*ij *_represent the Euclidean distances between the points, *i *and *j*, in the *n*- and *m*-dimensional spaces respectively.

The SOM results were obtained using the *SOM Toolbox*, which is a *Matlab*^® ^implementation [36,37]. Each training process consists of two phases. The following parameters were used. Initial learning rates equal to 0.5 (first phase) and 0.05 (second phase). Learning rates were controlled by an inverse-of-time function. The SOM neighborhood radius starts covering one fourth of the map size. The number of training epochs was equal to 10 times the number of map nodes (first phase). For the second phase it was equal to 4 times the number of training epochs in the first phase. A U-matrix depicts the distances between neighbouring map units by displaying a grey scale. In a label map a SOM node represents a class based on a majority voting strategy for the samples associated with this node. In case of a draw, the first class encountered is used. Empty nodes are not labeled. The Sammon's mapping analyses were implemented using the SOM Toolbox with 100 mapping iterations, iteration step size equal to 0.2 and the Euclidian distance.

For the interaction data set, the tool *inBuilder *was used to transform it into *interClust *input format. The cross-platform tools *interClust *and *inBuilder *are available for academic researchers on request from the authors.

Graphical outputs for the relaxation maps were obtained with the proprietary software *Statistica*^©^. Additional file 7 was created using *InterViewer *[40], which is freely available at [52]. Analyses were performed on a PC with a *Pentium*^® ^*4 *CPU.

## Authors' contributions

FA designed the study, implemented the relaxation non-linear mapping algorithm, performed analyses and wrote the manuscript. HW did the SOM and Sammon's mapping experiments and helped with the preparation of the manuscript. AC selected the interactome data, interpreted results and helped with the preparation of the manuscript.

## Supplementary Material

Additional File 1 **SOM frequency map for SRBCT data **It shows the distribution of samples, *X*(*Y*), over each node in Figure 5, where *X *represents the class label and *Y *stands for the number of Class *X *samples assigned to the corresponding node. Click here for file

Additional File 2 **3D visual display originating from relaxation non-linear mapping – SRBCT data **EWS, RMS and BL samples are represented by symbols '1', '2', '3' respectively. Click here for file

Additional File 3 **3D Sammon's mapping results – SRBCT data **Symbols "1", "2" and "3" represent classes EWS, RMS and BL respectively. Click here for file

Additional File 4 **SOM frequency map for Parkinson's disease data **It shows the distribution of samples, *X*(*Y*), over each node in Figure 8, where *X *represents the class label and *Y *stands for the number of Class *X *samples assigned to the corresponding node. Click here for file

Additional File 5 **3D Relaxation non-linear mapping of the Parkinson's disease model data **Parkinson's disease and Normal samples are identified by symbols '1' and '2' respectively. Click here for file

Additional File 6 **3D Sammon's mapping of the Parkinson's disease model data **Symbols "1" and "2" represent Parkinson's disease and Normal samples respectively. Click here for file

Additional File 7 **Examples of key hubs in the interactome **It depicts some of the hubs automatically isolated by the mapping algorithm, as well as a few nodes located near the centre of the map (F15A2.6, C34E10.5, C14B9.6). Click here for file

Additional File 8 **Description of protein cluster obtained from Figure **14 Click here for file

Additional File 9 **Leukaemia data set analysed in this paper **Log ratios are used to represent the expression levels. The last column shows the class labels. Click here for file

Additional File 10 **SRBCT data set analysed in this paper **Log ratios are used to represent the expression levels. The last column shows the class labels. Click here for file

Additional File 11 **Parkinson's disease data set analysed in this paper **Log ratios are used to represent the expression levels. The last column shows the class labels. Click here for file

Additional File 12 **Chromatin interaction network in *C. elegans ***The last column shows the connection strength in the graph. All connections are considered equally. Click here for file
