# Supplementary figures and images for: Non-linear mapping for exploratory data analysis in functional genomics
Source: BMC Bioinformatics. 2005 Jan 20;6:13. doi: 10.1186/1471-2105-6-13 (PMC548129; doi:10.1186/1471-2105-6-13)

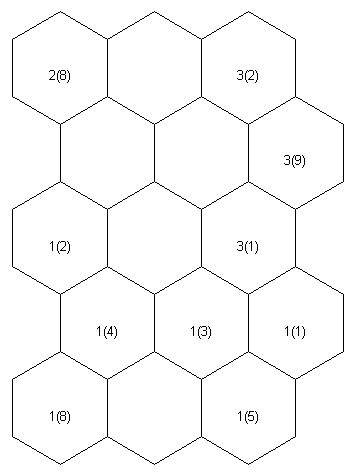

Supplement: Additional File 1 — SOM frequency map for SRBCT data It shows the distribution of samples, X(Y), over each node in Figure 5, where X represents the class label and Y stands for the number of Class X samples assigned to the corresponding node. [file 1471-2105-6-13-S1.bmp]

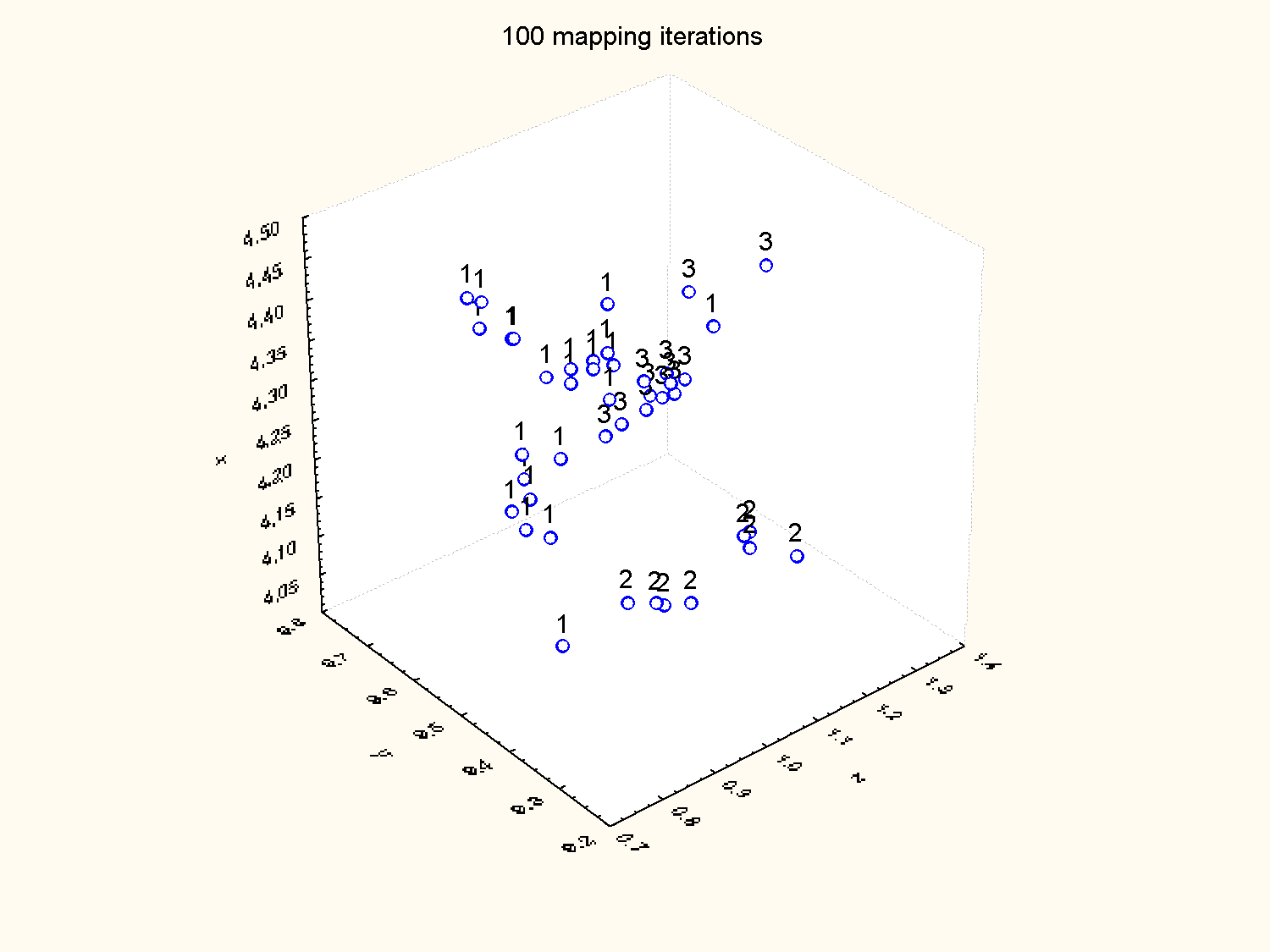

Supplement: Additional File 2 — 3D visual display originating from relaxation non-linear mapping – SRBCT data EWS, RMS and BL samples are represented by symbols '1', '2', '3' respectively. [file 1471-2105-6-13-S2.bmp]

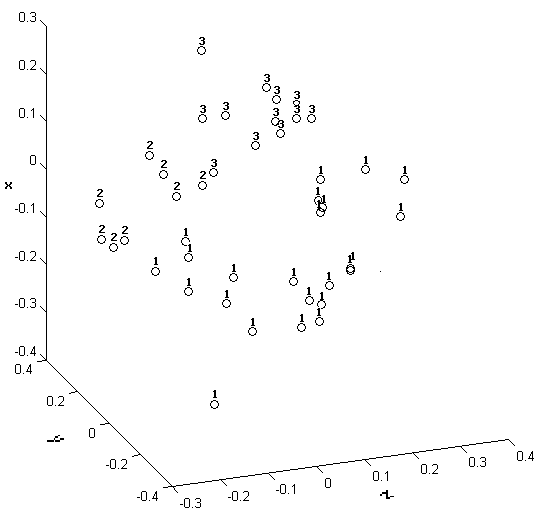

Supplement: Additional File 3 — 3D Sammon's mapping results – SRBCT data Symbols "1", "2" and "3" represent classes EWS, RMS and BL respectively. [file 1471-2105-6-13-S3.bmp]

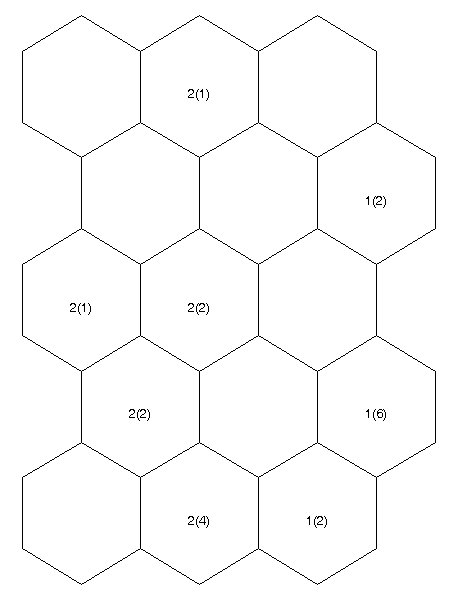

Supplement: Additional File 4 — SOM frequency map for Parkinson's disease data It shows the distribution of samples, X(Y), over each node in Figure 8, where X represents the class label and Y stands for the number of Class X samples assigned to the corresponding node. [file 1471-2105-6-13-S4.bmp]

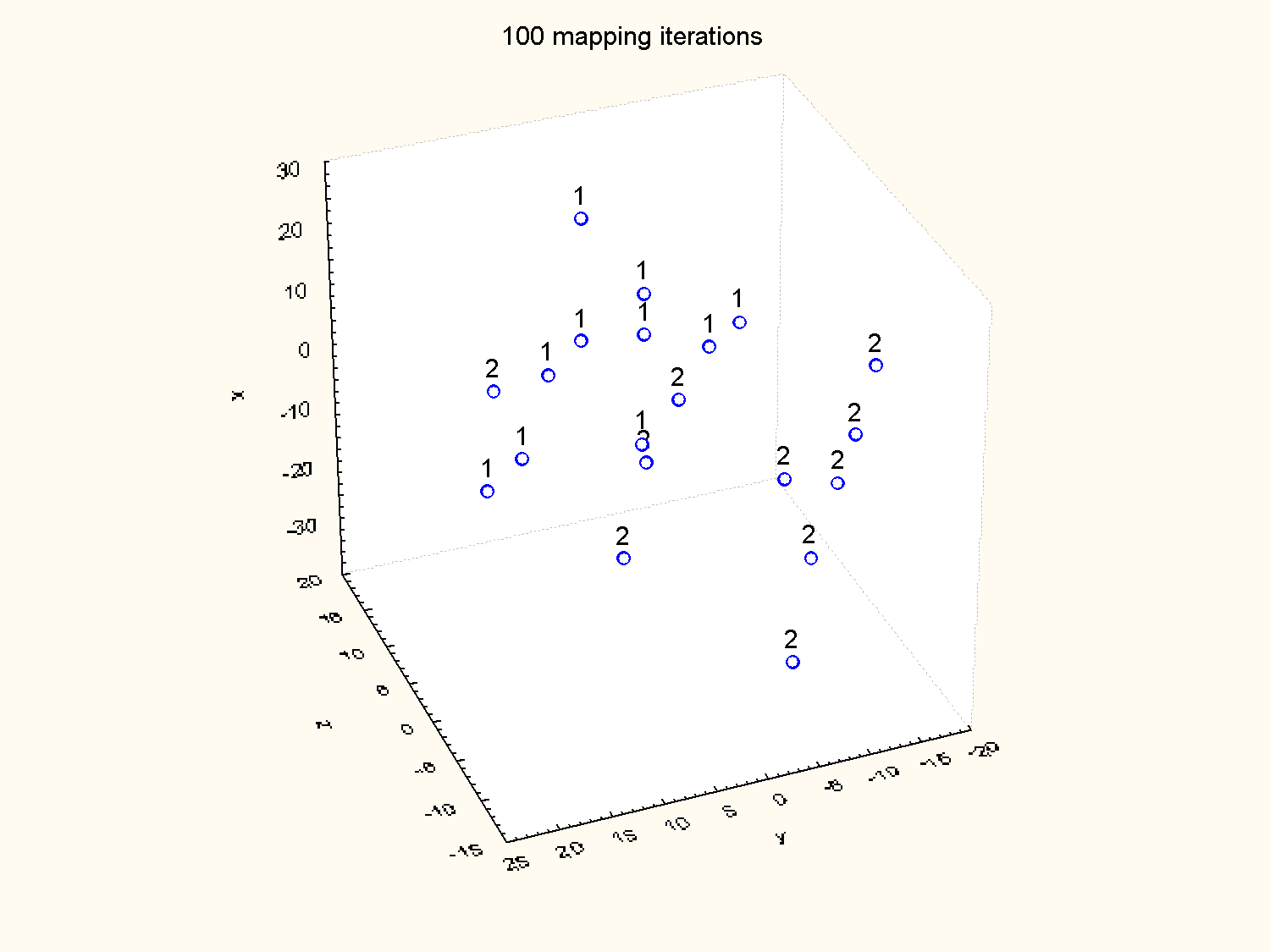

Supplement: Additional File 5 — 3D Relaxation non-linear mapping of the Parkinson's disease model data Parkinson's disease and Normal samples are identified by symbols '1' and '2' respectively. [file 1471-2105-6-13-S5.bmp]

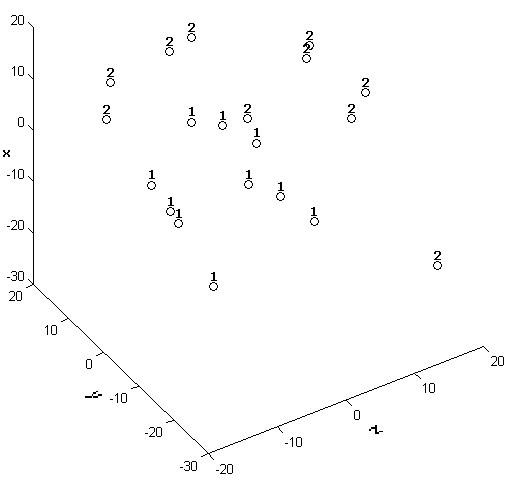

Supplement: Additional File 6 — 3D Sammon's mapping of the Parkinson's disease model data Symbols "1" and "2" represent Parkinson's disease and Normal samples respectively. [file 1471-2105-6-13-S6.bmp]

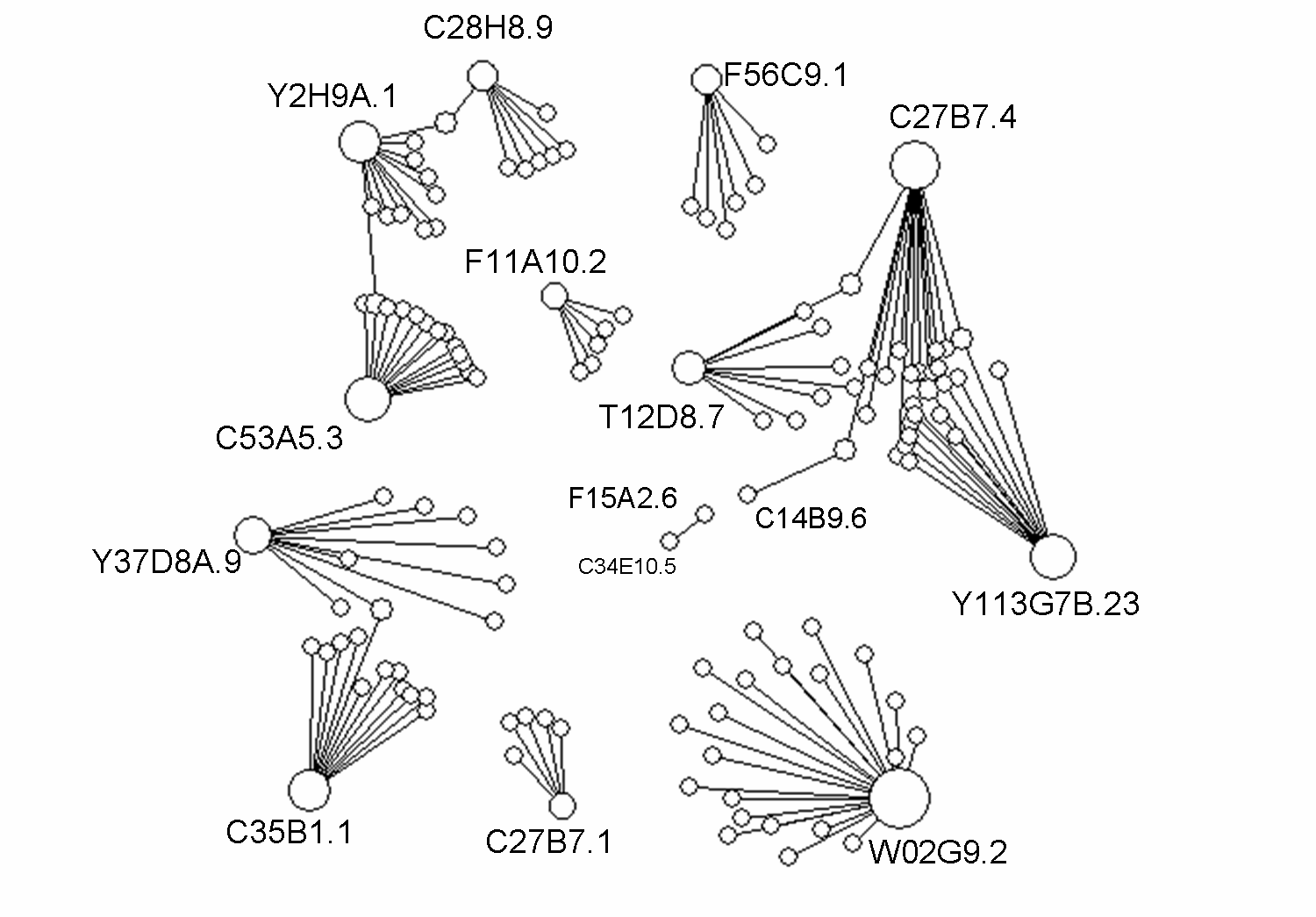

Supplement: Additional File 7 — Examples of key hubs in the interactome It depicts some of the hubs automatically isolated by the mapping algorithm, as well as a few nodes located near the centre of the map (F15A2.6, C34E10.5, C14B9.6). [file 1471-2105-6-13-S7.bmp]
