# Supplementary material for: Non-linear mapping for exploratory data analysis in functional genomics
Source: BMC Bioinformatics. 2005 Jan 20;6:13. doi: 10.1186/1471-2105-6-13 (PMC548129; doi:10.1186/1471-2105-6-13)
Supplement: Additional File 8 — Description of protein cluster obtained from Figure 14 [file 1471-2105-6-13-S8.pdf]

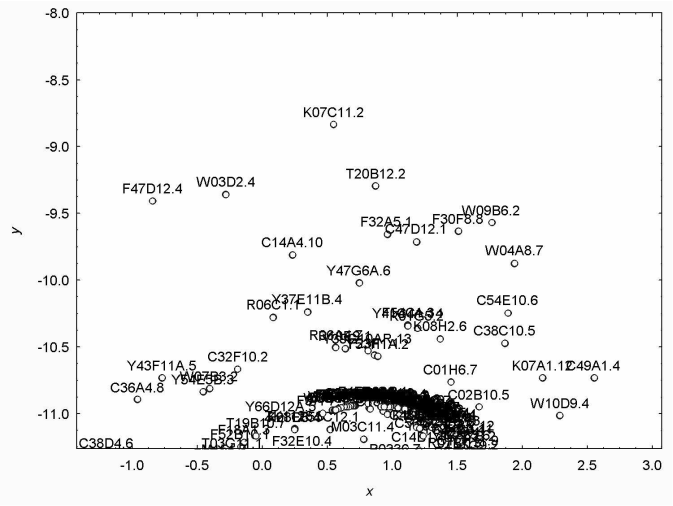

Gene name	Other name	Description
T20B12.2	Tbp-1	member of the holoenzyme Polymerase II
W09B6.2	Taf-6.1	member of the holoenzyme Polymerase II
W04A8.7	Taf-1	member of the holoenzyme Polymerase II
C14A4.10	Taf-13	member of the holoenzyme Polymerase II
F30F8.8	Taf-5	member of the holoenzyme Polymerase II
Y37E11B.4	Taf-2	member of the holoenzyme Polymerase II
C47D12.1	Trr-1	component of the SAGA/ADA histone acetyltransferase complex (interact II)
F32A5.1	Ada-2	component of the SAGA/ADA histone acetyltransferase complex (interact II)
W03D2.4	Pcn-1	
Y47G6A.6	pcaf-1	component of the PCAF histone acetyltransferase complex
R06C1.1	Hda-3	histone deacetylase enzyme
R06A4.7	Mes-2	component of a histone methyltransferase complex
F54C1.3	Mes-3	component of a histone methyltransferase complex
Y2H9A.1	Mes-4	component of a histone methyltransferase complex
Y54E5B.3		protein with a histone methyltransferase domain
Y43F11A.5		protein with a histone methyltransferase domain
K07C11.2	air-1	histone kinase
W07B3.2		similar to the holoenzyme Polymerase II uquitin enzyme
F47D12.4	hmg-1.2	component of the chromatin
C54E10.6		component of the chromatin
C38C10.5	rgr-1	mediator, interact directly with the holoenzyme Polymerase II
Y54E5B.3	let-49	mediator, interact directly with the holoenzyme Polymerase II
K08H2.6	hpl-1	chromatin organization modifier (methylated histones binding domain)
K01G5.2	hpl-2	chromatin organization modifier (methylated histones binding domain)
C01H6.7		bromodomain protein (acetylated histones binding domain)
C32F10.2	lin-35	Retinoblastoma binding protein
W10D9.4		histone-like transcription factor
